# *Phellodendron amurense* Leaf Extract Inhibits Rhabdovirus Infection by Targeting Early Stages of Viral Entry

**DOI:** 10.3390/pathogens15050491

**Published:** 2026-05-01

**Authors:** Su Yeon Kim, Taek-Kyun Lee, Tae-Jin Choi

**Affiliations:** 1 Department of Microbiology, School of Marine and Fisheries Sciences, Pukyong National University, Busan 48513, Republic of Korea; gsuyeon634@gmail.com; 2 Risk Assessment Research Center, Korea Institute of Ocean Science & Technology, Geoje 53201, Republic of Korea; tklee@kiost.ac.kr

**Keywords:** broad-spectrum antivirals, surrogate virus, VHSV, *Phellodendron amurense* leaf

## Abstract

RNA viruses exhibit high mutation rates, necessitating antivirals targeting conserved infection mechanisms. In this study, viral hemorrhagic septicemia virus (VHSV), a non-human pathogenic negative-sense RNA virus, was used as a surrogate model to enable high-throughput antiviral screening under reduced biosafety conditions. A recombinant VHSV expressing enhanced green fluorescent protein was used to screen 17,265 compounds, 2000 plant extracts, and 100 marine extracts. Among the candidates, the leaf extract of *Phellodendron amurense* Rupr. (PL extract) exhibited antiviral activity with low cytotoxicity (selectivity index ≈ 10). The extract reduced viral infectivity in a dose-dependent manner and showed cross-activity against snakehead rhabdovirus. Mechanistic analyses indicated that the PL extract acts primarily at early stages of infection. Virucidal assays demonstrated direct, time-dependent inactivation of viral particles, while pre-treatment reduced host cell susceptibility. Time-of-addition experiments confirmed that antiviral activity was restricted to early infection, suggesting interference with viral attachment or entry rather than intracellular replication. Fractionation revealed that activity was associated with the non-polar n-hexane fraction, implicating lipophilic compounds that may disrupt viral envelope integrity or membrane interactions. These findings suggest that *P. amurense* leaf extract is a promising candidate for broad-spectrum antivirals targeting conserved entry processes in enveloped RNA viruses.

## 1. Introduction

Viruses are obligate intracellular parasites that rely on host cellular machinery for replication and actively modulate host processes during infection [1]. RNA viruses, in particular, exhibit high mutation rates and genetic diversity due to rapid replication, limited proofreading capacity, and frequent genetic exchange through recombination or reassortment [2,3]. These characteristics facilitate viral adaptation and drive the emergence and re-emergence of RNA virus-associated diseases, including those caused by influenza virus, Ebola virus, and members of the family *Rhabdoviridae*.

Vaccination and antiviral therapeutics remain the primary strategies for controlling viral infections [4]. However, vaccine efficacy can be compromised by rapid viral evolution, highlighting the need for antiviral agents that target conserved viral or host mechanisms. Advances in understanding viral replication and host–virus interactions have therefore shifted antiviral discovery toward the identification of broad-spectrum agents [5,6]. In this context, compound- and natural extract-based screening approaches have been widely used to identify novel antiviral candidates [7,8,9].

The development of antivirals against human RNA viruses is often constrained by biosafety requirements, as many clinically relevant viruses require biosafety level (BSL)-3 or higher containment. To address this limitation, surrogate virus systems that can be handled under lower biosafety conditions are increasingly employed for large-scale screening. Such systems improve experimental accessibility while preserving biological relevance through shared genetic and mechanistic features with target viruses [10,11,12,13].

Viral hemorrhagic septicemia virus (VHSV), a fish-pathogenic negative-sense single-stranded RNA virus belonging to the family *Rhabdoviridae* and order *Mononegavirales*, represents a suitable surrogate model. Its genome comprises six genes (N, P, M, G, NV, and L), and its replication follows the conserved polymerase-dependent transcriptional strategy characteristic of non-segmented negative-sense RNA viruses [14,15,16,17]. These features are shared with several human-pathogenic viruses, including Ebola virus, measles virus, and rabies virus, supporting the relevance of VHSV as a model for studying conserved antiviral targets [18,19,20]. In addition to its utility as a surrogate, VHSV is of significant economic importance in aquaculture due to its global distribution, broad host range, and impact on fish health [21,22,23,24,25,26].

In this study, we employed a recombinant VHSV expressing enhanced green fluorescent protein (rVHSV–eGFP) as a high-throughput screening platform to evaluate a large library of synthetic compounds and natural extracts [27]. This system enables rapid and quantitative detection of viral infection through fluorescence-based readouts. Using this approach, we identified the leaf extract of *Phellodendron amurense* as a candidate antiviral agent with a favorable selectivity index. Its antiviral activity was further characterized through stage-specific assays to elucidate its mechanism of action, and its broader antiviral potential was evaluated against snakehead rhabdovirus (SHRV), another member of the genus *Novirhabdovirus*.

## 2. Materials and Methods

We followed the procedures for mass screening of anti-RNA virus substances by rVHSV–eGFP are described previously with minor modifications for the screening of additional chemical compounds and plant extracts.

### 2.1. Cell Lines and Viruses

*Epithelioma papulosum cyprini* (EPC) cells and rVHSV–eGFP were kindly provided by Prof. Ki-Hong Kim (Department of Aquatic Life Medicine, Pukyong National University, Republic of Korea). EPC cells were maintained in Leibovitz’s L-15 medium (Gibco, Thermo Fisher Scientific, Grand Island, NY, USA) supplemented with 10% fetal bovine serum (FBS; Gibco, Thermo Fisher Scientific, USA) and penicillin–streptomycin (100 U/mL penicillin and 100 µg/mL streptomycin) at 28 °C. rVHSV was propagated in EPC cells cultured in L-15 medium supplemented with 2% FBS and antibiotics at 15 °C. When cytopathic effects (CPE) were observed, culture supernatants were collected, clarified by centrifugation (1500× *g*, 5 min), filtered through a 0.45 μm Minisart CA syringe filter (Sartorius, Göttingen, Germany), and stored at −80 °C. Viral titers were determined by plaque assay. For plaque assays, virus-containing supernatants were serially diluted tenfold in L-15 medium supplemented with 2% FBS and antibiotics. EPC cells (1.2 × 10^6^ cells/well) were seeded in 6-well plates and incubated to form confluent monolayers. After removal of the medium, each well was inoculated with 200 µL of diluted virus and supplemented with 1.8 mL of medium, followed by incubation at 15 °C for 2 h to allow viral adsorption. The inoculum was then removed, and cells were overlaid with 2 mL of L-15 medium containing 0.8% agar, 2% FBS, and antibiotics. Plates were incubated at 15 °C for 7 days. Plaques were fixed with 4% paraformaldehyde (GeneAll, Seoul, Republic of Korea) at room temperature for 5 h. Agar overlays were carefully removed, and cell monolayers were stained with 10% crystal violet overnight. After washing, plaques were counted.

### 2.2. High-Throughput Screening Assay

#### 2.2.1. Screening Materials

A total of 17,265 compounds, 2000 plant-derived extracts, and 100 marine-derived extracts were included in the screening assay. Compounds were obtained from the Korea Chemical Bank (KCB), while plant and marine extracts were provided by the National Institute of Biological Resources (NIBR) and the National Marine Biodiversity Institute of Korea (MABIK), respectively. Compounds were supplied as 5 mM stock solutions in 96-well plates and used directly for screening. Plant and marine extracts (approximately 20 mg of dry weight) were dissolved in 10% dimethyl sulfoxide (DMSO) to prepare stock solutions at a concentration of 20 mg/mL.

#### 2.2.2. Screening Procedure

High-throughput screening was performed in 96-well plates by dispensing 5 µL of each test substance into individual wells. EPC cells (1.0 × 10^5^ cells/well) were mixed with rVHSV at a multiplicity of infection (MOI) of 0.01 in a sterile flask and gently stirred at 200 rpm using a magnetic stirrer (LabTron, Seoul, Republic of Korea). The cell–virus mixture (195 µL) was then dispensed into each well using a MicroFill microplate dispenser (BioTek, Winooski, VT, USA), resulting in final concentrations of 125 µM for compounds and 500 µg/mL for extracts. Plates were incubated at 28 °C for 24 h to allow cell attachment and monolayer formation, followed by incubation at 15 °C for 7 days. At the end of the infection period, cytopathic effects (CPE) were assessed using an inverted microscope (Motic, Xiamen, China), and enhanced green fluorescent protein (eGFP) fluorescence was measured using a Varioskan LUX multimode microplate reader (Thermo Fisher Scientific, USA) at excitation/emission wavelengths of 490/516 nm. For secondary screening, candidates showing consistent inhibition of virus-induced CPE and reduction of eGFP fluorescence in the primary screen were selected and evaluated across concentration ranges of 25–125 µM for compounds and 62.5–500 µg/mL for extracts. EPC cells (1.0 × 10^5^ cells/well) were seeded into 96-well plates and incubated to form monolayers. After removal of the culture medium, cells were treated with 5 µL of test substances at the indicated concentrations, together with 195 µL of L-15 medium supplemented with 2% FBS and antibiotics and containing rVHSV (MOI = 0.01). Following incubation at 15 °C for 7 days post-infection, antiviral activity was evaluated based on the inhibition of CPE using an inverted microscope.

### 2.3. Plant Extract

The leaf extract of *Phellodendron amurense* Rupr. (PL extract), identified as a candidate in the screening assay, was used for further evaluation. The extract was obtained from the National Institute of Biological Resources (NIBR, Incheon, Republic of Korea; resource number: NIBRGR0000687085) as dried leaf material.

### 2.4. Cytotoxicity Assay

Cytotoxicity was evaluated using the Ez-Cytox assay kit (DoGenBio, Seoul, Republic of Korea) according to the manufacturer’s instructions. EPC cells (1.0 × 10^5^ cells/well) were seeded in 96-well plates and incubated to allow monolayer formation. The PL extract (5 µL) was added to achieve final concentrations of 25, 50, 100, 200, and 400 µg/mL, followed by the addition of 195 µL of L-15 medium supplemented with 10% fetal bovine serum (FBS) and penicillin–streptomycin (100 U/mL and 100 µg/mL, respectively). Control wells received 5 µL of 10% dimethyl sulfoxide (DMSO) and 195 µL of the same medium. All treatments were performed in triplicate. After incubation at 28 °C for 48 h, the medium was removed, and cells were washed with 200 µL of phosphate-buffered saline (PBS, pH 7.4). Subsequently, 100 µL of L-15 medium supplemented with 10% FBS and antibiotics and 10 µL of water-soluble tetrazolium (WST) reagent were added to each well. Plates were incubated at 28 °C for 3 h, and absorbance at 450 nm was measured using a Varioskan LUX multimode microplate reader (Thermo Fisher Scientific, Waltham, MA, USA). Cell viability (%) was calculated as (A_sample_ − A_blank_)/(A_control_ − A_blank_) × 100. The 50% cytotoxic concentration (CC_50_) was determined by fitting dose–response curves using a four-parameter log-logistic (4PL) model implemented in R (version 4.4.3).

### 2.5. Antiviral Activity Assay

Antiviral activity against rVHSV was evaluated by quantifying viral titers using a plaque assay. EPC cells (1.0 × 10^5^ cells/well) were seeded in 96-well plates and incubated to allow monolayer formation. After removal of the culture medium, cells were treated with 5 µL of PL extract to achieve final concentrations of 10, 25, 50, 100, and 200 µg/mL, together with 195 µL of L-15 medium supplemented with 2% fetal bovine serum (FBS) and penicillin–streptomycin (100 U/mL and 100 µg/mL, respectively) and containing rVHSV at a multiplicity of infection (MOI) of 0.01. Plates were incubated at 15 °C for 7 days, after which culture supernatants were collected for plaque assay as described in Section 2.1. Viral infectivity (%) was calculated as (PFU_sample_/PFU_control_) × 100. The 50% inhibitory concentration (IC_50_) was determined by fitting dose–response curves using a four-parameter log-logistic (4PL) model implemented in R (version 4.4.3).

### 2.6. RNA Extraction and RT-qPCR

Total RNA was extracted using the HiYield Total RNA Mini Kit (Blood/Bacteria/Cultured Cells; RBC Bioscience, Taipei, Taiwan) according to the manufacturer’s instructions. RNA concentration and purity were determined using a NanoDrop OneC spectrophotometer (Thermo Fisher Scientific, Waltham, MA, USA). To remove genomic DNA contamination, 170 ng of total RNA was treated with 1 U of DNase I (Thermo Fisher Scientific, Waltham, MA) and subsequently reverse-transcribed into cDNA using Reverse Transcriptase Premix (Oligo d(T)_15_) (ELPIS-Biotech, Daejeon, Republic of Korea). Quantitative real-time PCR (RT-qPCR) was performed in a 20 µL reaction volume containing 10 µL of AccuPower 2× GreenStar qPCR Master Mix (Bioneer, Daejeon, Republic of Korea), primers at a final concentration of 0.3 µM each (Table 1), 1 µL of cDNA template, and nuclease-free water. Amplification was carried out using a QuantStudio 6 Flex Real-Time PCR System (Thermo Fisher Scientific, Waltham, MA, USA) under the following conditions: initial denaturation at 95 °C for 10 min, followed by 40 cycles of 95 °C for 15 s, 58 °C for 15 s, and 72 °C for 20 s, with a subsequent melting curve analysis.

### 2.7. Evaluation of Antiviral Mechanism

#### 2.7.1. Virucidal Assay

EPC cells (3.0 × 10^5^ cells/well) were seeded in 48-well plates and incubated to allow monolayer formation. The PL extract was mixed with rVHSV (7.5 × 10^5^ PFU/mL) to achieve a final concentration of 100 µg/mL and incubated at 15 °C for 15, 30, or 60 min. As a control, rVHSV without PL extract (virus-only control) was incubated under the same conditions.

To evaluate direct virucidal activity while minimizing the effects of residual extract, the virus–extract mixture was diluted 100-fold with L-15 medium supplemented with 2% fetal bovine serum (FBS) and penicillin–streptomycin (100 U/mL and 100 µg/mL, respectively), thereby reducing the extract concentration to a level with minimal impact on viral replication. Subsequently, 400 µL of the diluted mixture was inoculated into each well of EPC cells.

Following inoculation, cells were incubated at 15 °C for 48 h and then harvested for RNA extraction. RNA extraction and quantitative real-time PCR (RT-qPCR) analyses were performed as described in Section 2.6.

#### 2.7.2. Pre-Treatment Assay

EPC cells (3.0 × 10^5^ cells/well) were seeded in 48-well plates and incubated to allow monolayer formation. The PL extract was diluted in L-15 medium supplemented with 2% fetal bovine serum (FBS) and penicillin–streptomycin (100 U/mL and 100 µg/mL, respectively) and added to each well to achieve a final concentration of 100 µg/mL in a total volume of 400 µL. Cells were pretreated with the extract at 28 °C for 12 or 24 h, while control cells without PL extract (untreated control) were maintained under the same conditions.

Following pretreatment, the medium was removed, and cells were washed with phosphate-buffered saline (PBS) prior to infection with rVHSV at a multiplicity of infection (MOI) of 0.01. Infected cells were incubated at 15 °C for 48 h and subsequently harvested for RNA extraction. RNA extraction and quantitative real-time PCR (RT-qPCR) analyses were performed as described in Section 2.6.

#### 2.7.3. Time-of-Addition Assay

EPC cells (3.0 × 10^5^ cells/well) were seeded in 48-well plates and incubated to allow monolayer formation, followed by infection with rVHSV at a multiplicity of infection (MOI) of 0.01. The PL extract was added at 0, 2, 4, 6, and 8 h post-infection (hpi) to achieve a final concentration of 100 µg/mL in a total volume of 400 µL.

Infected cells without PL extract (virus-only control) were maintained under the same conditions. At 2 h after each treatment, the medium was removed, and cells were washed with phosphate-buffered saline (PBS). Cells were then incubated at 15 °C for 48 h and subsequently harvested for RNA extraction. RNA extraction and quantitative real-time PCR (RT-qPCR) analyses were performed as described in Section 2.6.

### 2.8. Fractionation of Plant Extract

For fractionation, 300 g of *P. amurense* leaves were collected from the Hallasan Eco-forest (Jeju Island, Republic of Korea). Extraction was performed at the Nakdonggang National Institute of Biological Resources (NNIBR, Sangju, Republic of Korea), and the material was provided as a dried extract (24.37 g). The crude extract (3 g) was dissolved in 100 mL of 5% ethanol in water and subjected to liquid–liquid partitioning with n-hexane, ethyl acetate, and n-butanol in order of increasing polarity. Each solvent (100 mL) was applied twice during fractionation.

Organic fractions were concentrated using a rotary evaporator, while the residual aqueous fraction was lyophilized. All fractions were subsequently used for antiviral evaluation.

EPC cells (1.0 × 10^5^ cells/well) were seeded in 96-well plates and incubated to allow monolayer formation. Cells were then treated with each fraction and the crude extract at concentrations of 1–200 µg/mL. Following incubation at 15 °C for 7 days post-infection, cytopathic effects (CPE) were assessed using an inverted microscope. Fractions showing inhibitory effects on rVHSV-induced CPE were further evaluated by cytotoxicity and plaque assays to determine CC_50_ and IC_50_ values.

### 2.9. Antiviral Activity Against SHRV

The antiviral activity of the PL extract against snakehead rhabdovirus (SHRV), a member of the genus *Novirhabdovirus* within the family *Rhabdoviridae*, was further evaluated in EPC cells. SHRV was kindly provided by Prof. Ki-Hong Kim (Department of Aquatic Life Medicine, Pukyong National University, Republic of Korea) and propagated in EPC cells maintained in L-15 medium supplemented with 2% fetal bovine serum (FBS) and penicillin–streptomycin (100 U/mL and 100 µg/mL, respectively) at 28 °C. Virus-containing culture supernatants exhibiting cytopathic effects (CPE) were collected, clarified by centrifugation (1500× *g*, 5 min), filtered through a 0.45 µm CA syringe filter (Sartorius, Göttingen, Germany), and stored at −80 °C.

Antiviral activity against SHRV was assessed by quantifying viral titers using a plaque assay. EPC cells (1.0 × 10^5^ cells/well) were seeded in 96-well plates and incubated to allow monolayer formation. After removal of the culture medium, cells were treated with 5 µL of PL extract to achieve final concentrations of 2.5–200 µg/mL, together with 195 µL of L-15 medium supplemented with 2% FBS and antibiotics and containing SHRV at a multiplicity of infection (MOI) of 0.01. Following incubation at 28 °C for 3 days, culture supernatants were collected for plaque assay.

For SHRV plaque assays, virus-containing supernatants were serially diluted tenfold in L-15 medium supplemented with 2% FBS and antibiotics. EPC cells (1.2 × 10^6^ cells/well) were seeded in 6-well plates and incubated to form confluent monolayers. After removal of the medium, each well was inoculated with 200 µL of diluted virus and incubated at 28 °C for 2 h to allow viral adsorption. The inoculum was then removed, and cells were overlaid with 2 mL of L-15 medium containing 0.8% agar, 2% FBS, and antibiotics. Plates were incubated at 28 °C for 3 days, followed by fixation and staining as described in Section 2.1.

## 3. Results

### 3.1. Identification of PL Extract as an Antiviral Candidate

**Figure 1:**
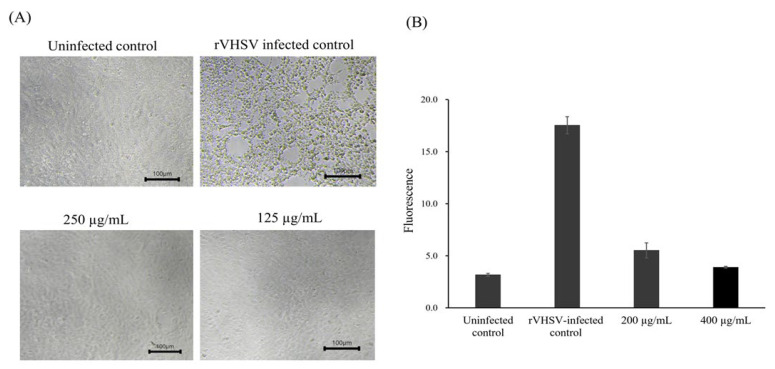
Inhibitory effects of *Phellodendron amurense* leaf extract (PL extract) on rVHSV-induced cytopathic effects (CPE) and eGFP expression in EPC cells. (**A**) Representative microscopic images of EPC cells showing uninfected control, rVHSV-infected control (without treatment), and cells treated with PL extract (125 and 250 µg/mL). Treatment with PL extract reduced rVHSV-induced cytopathic effects compared with the infected control. All images were acquired at the same magnification (100×). Scale bars represent 100 µm. (**B**) eGFP fluorescence in rVHSV-infected cells treated with PL extract (200 and 400 µg/mL). Fluorescence intensity was measured at excitation/emission wavelengths of 490/516 nm. Data are presented as mean ± SEM from three independent biological replicates.

Primary screening at 125 µM for compounds and 500 µg/mL for extracts identified 18 compounds and 8 extracts with antiviral activity against rVHSV, based on inhibition of cytopathic effects (CPE) and reduced eGFP expression. In secondary screening, these candidates were evaluated across concentration ranges of 25–125 µM for compounds and 62.5–500 µg/mL for extracts, resulting in the selection of three compounds and one extract for further analysis. Among these, the leaf extract of *P. amurense* Rupr. (PL extract) markedly inhibited rVHSV-induced CPE at concentrations ≥ 125 µg/mL (Figure 1A). Consistently, rVHSV-induced eGFP expression was substantially reduced in cells treated with the PL extract at 200 and 400 µg/mL (Figure 1B).

### 3.2. Antiviral Efficacy and Selectivity

#### 3.2.1. Cytotoxicity

**Figure 2:**
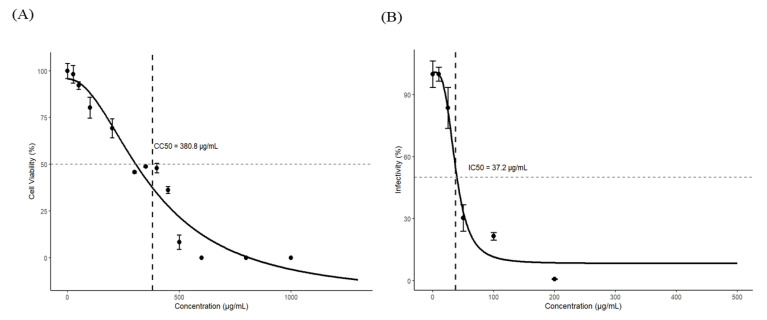
Cytotoxicity and antiviral activity of *Phellodendron amurense* leaf extract (PL extract) against rVHSV in EPC cells. (**A**) Cell viability of EPC cells treated with increasing concentrations of PL extract. Cell viability (%) was calculated as (A_sample_ − A_blank_)/(A_control_ − A_blank_) × 100. (**B**) Inhibition of rVHSV infectivity by PL extract, determined by plaque assay. Viral infectivity (%) was calculated as the percentage of plaque-forming units (PFU) in treated samples relative to the virus-infected control. Dashed lines indicate the 50% response level (horizontal) and CC_50_ (**A**) or IC_50_ (**B**) (vertical). Data are presented as mean ± SEM from three independent biological replicates (*n* = 3).

The cytotoxicity of the PL extract in EPC cells was evaluated by measuring cell viability after 48 h of treatment. As shown in Figure 2A, cell viability remained above 80% at concentrations up to 100 µg/mL, indicating low cytotoxicity. In contrast, treatment at 400 µg/mL reduced cell viability to below 50%. The 50% cytotoxic concentration (CC_50_) of the PL extract was estimated to be 380.79 ± 75.27 µg/mL.

#### 3.2.2. Antiviral Activity

The antiviral activity of the PL extract against rVHSV was evaluated by plaque assay through quantification of infectious viral particles in culture supernatants. As shown in Figure 2B, the PL extract inhibited rVHSV infectivity in a dose-dependent manner. Treatment with 200 µg/mL reduced viral infectivity to <1% of the control (>99% inhibition). At 50 and 100 µg/mL, viral infectivity was reduced by 69.61% and 78.46%, respectively. The 50% inhibitory concentration (IC_50_) was determined to be 37.2 ± 5.66 µg/mL. The selectivity index (SI), calculated as CC_50_/IC_50_, was approximately 10.24.

### 3.3. Mode of Antiviral Action

**Figure 3:**
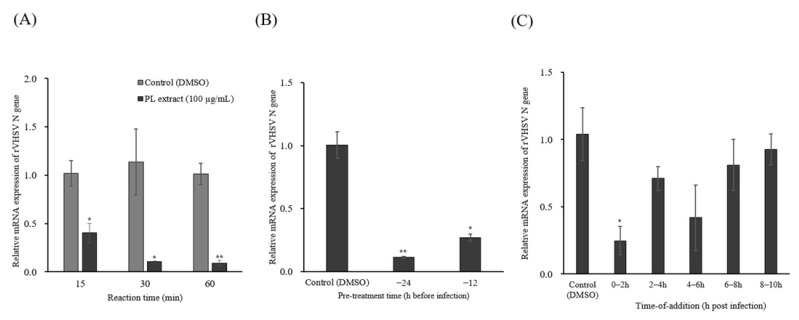
Stage-specific antiviral effects of *Phellodendron amurense* leaf extract (PL extract) on rVHSV infection in EPC cells. (**A**) Virucidal assay: rVHSV was incubated with PL extract (100 µg/mL) for 15–60 min at 15 °C prior to infection of EPC cells (MOI = 0.01). (**B**) Pre-treatment assay: EPC cells were treated with PL extract (100 µg/mL) for 12 or 24 h prior to infection. (**C**) Time-of-addition assay: PL extract (100 µg/mL) was added at 0–8 h post-infection (hpi). Relative mRNA expression of the rVHSV N gene was quantified at 2 days post-infection by quantitative real-time PCR (RT-qPCR) and normalized to β-actin. Data are presented relative to the virus-infected control. Data are presented as mean ± SEM from three independent biological replicates (*n* = 3). Statistical significance was determined using an unpaired Student’s *t*-test (* *p* < 0.05, ** *p* < 0.01).

The mode of antiviral action of the PL extract against rVHSV was investigated using virucidal, pre-treatment, and time-of-addition assays. A final concentration of 100 µg/mL was used in all experiments. In the virucidal assay, rVHSV was incubated with the PL extract for 15, 30, or 60 min prior to infection. Relative *N* gene mRNA levels were reduced in all treatment groups compared with the untreated virus-infected control, with greater inhibition observed at longer incubation times (Figure 3A). In the pre-treatment assay, EPC cells were treated with the PL extract for 12 or 24 h prior to infection. Pre-treatment reduced *N* gene mRNA levels to approximately 0.402 and 0.114 at 12 and 24 h, respectively (Figure 3B). In the time-of-addition assay, the PL extract was added at 0, 2, 4, 6, and 8 h post-infection (hpi). Significant inhibition was observed only when the extract was applied at 0–2 hpi, reducing *N* gene mRNA levels to approximately 0.25, whereas treatment at later time points did not result in statistically significant reductions (Figure 3C).

### 3.4. Antiviral Activity of Fractionated Extracts

**Figure 4:**
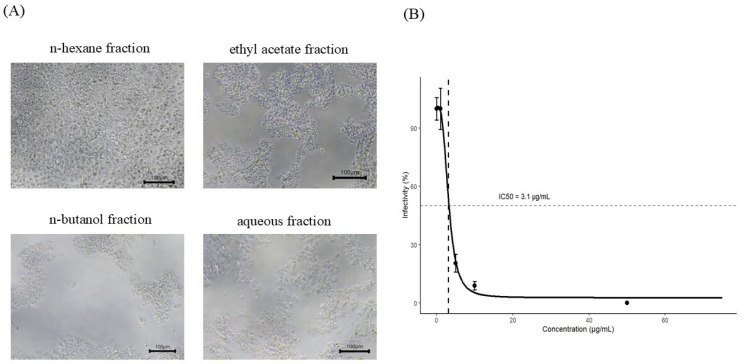
Screening of solvent fractions and antiviral activity of the n-hexane fraction of *Phellodendron amurense* leaf extract (PL extract) against rVHSV in EPC cells. (**A**) Representative microscopic images of EPC cells infected with rVHSV and treated with solvent fractions of PL extract (n-hexane, ethyl acetate, n-butanol, and aqueous fractions) at 25 µg/mL. The n-hexane fraction selectively reduced rVHSV-induced cytopathic effects (CPE) compared with the virus-infected control. All images were acquired at the same magnification (100×). Scale bars represent 100 µm. (**B**) Inhibition of rVHSV infectivity by the n-hexane fraction, determined by plaque assay. Viral infectivity (%) was calculated as the percentage of plaque-forming units (PFU) in treated samples relative to the virus-infected control. In (**B**), dashed lines indicate the 50% response level (horizontal) and IC_50_ (vertical). Data are presented as mean ± SEM from three independent biological replicates (*n* = 3).

EPC cells infected with rVHSV were treated with n-hexane, ethyl acetate, n-butanol, and aqueous fractions at concentrations ranging from 1 to 200 µg/mL. Among these, only the n-hexane fraction inhibited rVHSV-induced cytopathic effects (CPE), whereas the other fractions showed CPE comparable to the virus-infected control. As shown in Figure 4A, antiviral activity was observed exclusively in the n-hexane fraction, with detectable effects at concentrations as low as 25 µg/mL. The crude extract and the n-hexane fraction were further evaluated by plaque and cytotoxicity assays. The IC_50_ values were 16.3 ± 0.7 µg/mL for the crude extract and 3.1 ± 0.62 µg/mL for the n-hexane fraction (Figure S1 and Figure 4B). The CC_50_ value of the n-hexane fraction was 110 ± 2.85 µg/mL, whereas that of the crude extract was estimated to be 295.28 µg/mL by interpolation (Figure S2). Accordingly, the n-hexane fraction exhibited a higher selectivity index (SI) than the crude extract (Table 2).

### 3.5. Cross-Antiviral Activity Against SHRV

**Figure 5:**
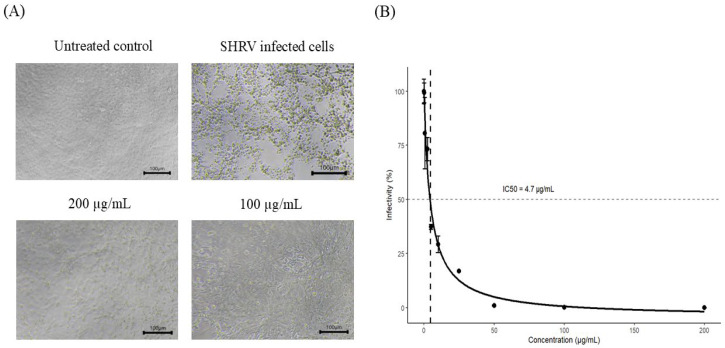
Antiviral activity of *Phellodendron amurense* leaf extract (PL extract) against snakehead rhabdovirus (SHRV) in EPC cells. (**A**) Representative microscopic images of EPC cells showing untreated control, SHRV-infected control (without treatment), and cells treated with PL extract (100 and 200 µg/mL). PL extract treatment reduced SHRV-induced cytopathic effects (CPE) compared with the virus-infected control. All images were acquired at the same magnification (100×). Scale bars represent 100 µm. (**B**) Inhibition of SHRV infectivity by PL extract, determined by plaque assay. Viral infectivity (%) was calculated as the percentage of plaque-forming units (PFU) in treated samples relative to the virus-infected control. In (**B**), dashed lines indicate the 50% response level (horizontal) and IC_50_ (vertical). Data are presented as mean ± SEM from three independent biological replicates (*n* = 3).

The PL extract markedly inhibited SHRV-induced cytopathic effects (CPE) at concentrations ≥ 100 µg/mL (Figure 5A). Plaque assay of culture supernatants demonstrated that the extract suppressed SHRV infectivity in a dose-dependent manner. Treatment with 100 µg/mL reduced viral titers to <0.1% of the virus-infected control (≥99.9% inhibition). The 50% inhibitory concentration (IC_50_) of the PL extract against SHRV was 4.664 ± 1.15 µg/mL (Figure 5B). The selectivity index (SI), calculated as CC_50_/IC_50_, was approximately 81.64.

## 4. Discussion

In this study, a high-throughput screening approach using recombinant viral hemorrhagic septicemia virus (rVHSV) expressing eGFP as a surrogate RNA virus model enabled efficient identification of antiviral candidates under reduced biosafety requirements. Through sequential screening, the leaf extract of *P. amurense* Rupr. (PL extract) was identified based on its ability to suppress rVHSV-induced cytopathic effects (CPE) and eGFP expression (Figure 1A,B). Subsequent analyses demonstrated that the PL extract reduced viral infectivity in a dose-dependent manner, as indicated by decreased plaque-forming unit (PFU) levels, with minimal cytotoxicity at concentrations up to 200 µg/mL (Figure 2A,B). The resulting selectivity index (SI ≈ 10.23) meets the commonly accepted threshold (SI ≥ 10) for promising antiviral agents, supporting the potential of the PL extract as an effective antiviral candidate [30,31,32].

Although *P. amurense* has been extensively studied for its antioxidant, anti-inflammatory, and antimicrobial properties, antiviral activity has primarily been associated with bark-derived extracts [33,34,35]. Previous studies have reported antiviral effects of bark extracts against herpes simplex virus type 1 (HSV-1), vesicular stomatitis virus (VSV), and influenza A virus subtypes [36,37]. In contrast, the antiviral activity of leaf-derived extracts remains poorly characterized. The present findings therefore expand the known bioactivity of *P. amurense* and suggest that leaf-derived constituents represent an underexplored source of antiviral compounds.

Identifying the stage(s) of the viral life cycle targeted by antiviral agents is essential for understanding their mechanisms of action [38,39]. In this study, stage-specific antiviral effects were evaluated by quantifying relative mRNA levels of the rVHSV nucleoprotein (*N*) gene using RT-qPCR. The *N* protein plays a critical role in viral replication and transcription [40,41] and is abundantly expressed due to the transcriptional gradient characteristic of rhabdoviruses [42,43]. Accordingly, *N* gene expression was used as a sensitive indicator of viral replication activity.

The PL extract exhibited antiviral activity predominantly during the early stages of infection. In the virucidal assay, *N* gene expression decreased in a time-dependent manner following incubation of the virus with the extract, indicating direct viral inactivation (Figure 3A). Similarly, pre-treatment of host cells resulted in a time-dependent reduction in viral RNA levels (Figure 3B), suggesting that the extract may modulate host cell susceptibility or interfere with early infection processes. In contrast, no significant inhibition was observed when the extract was applied during later stages of infection. Consistently, time-of-addition experiments showed that the antiviral effects were restricted to the early phase (0–2 h post-infection), indicating that the PL extract primarily interferes with early stages of infection rather than intracellular replication (Figure 3C). Viral entry and membrane fusion are critical steps in the viral life cycle and are widely recognized as promising targets for antiviral development [44]. However, our current data do not distinguish between inhibition of viral attachment and membrane fusion. Further studies, including virus-binding assays and fusion/entry assays, are required to clarify whether the PL extract inhibits viral attachment or membrane fusion processes.

Fractionation of the PL extract further supported this mechanism. Among the solvent fractions, only the n-hexane fraction inhibited rVHSV-induced CPE (Figure 4A), indicating that the antiviral activity is associated with low-polarity compounds. Lipophilic molecules are known to interact with viral lipid envelopes and disrupt membrane integrity or fusion processes in enveloped viruses [45,46]. Notably, *P. amurense* is known to contain bioactive isoquinoline alkaloids, including berberine, palmatine, and jatrorrhizine, as well as limonoid compounds. Several of these constituents, particularly berberine and limonoids, have been reported to exhibit antiviral activity against a range of viruses in previous studies [47,48]. These findings support the possibility that lipophilic constituents contribute to the observed antiviral effects, although further studies are required to identify the specific active compounds responsible. Consistent with this, recent reports have suggested that lipophilic phytochemicals, including terpenoids and certain alkaloids, may contribute to antiviral activity, potentially through interactions with viral membranes that affect envelope stability and infectivity [49,50]. The SI value of the PL extract used for initial screening was 10.24, whereas the extract used for fractionation exhibited a higher SI value of 18.1. Although the same ethanol extraction method was applied, this difference may reflect variability in the plant material, such as seasonal variation. Further studies are needed to determine the optimal harvesting conditions for maximizing antiviral activity. The n-hexane fraction exhibited a lower IC_50_ than the crude extract, indicating enhanced antiviral potency (Figure 4B), although a reduction in CC_50_ suggested increased cytotoxicity. Despite this, the higher selectivity index of the n-hexane fraction indicates improved antiviral specificity (Table 2). These findings suggest that lipophilic compounds within the n-hexane fraction are likely responsible for the observed antiviral effects; however, identification of the active constituents remains necessary.

Importantly, the PL extract also demonstrated strong antiviral activity against snakehead rhabdovirus (SHRV), another member of the genus Novirhabdovirus. The extract effectively inhibited SHRV-induced CPE (Figure 5A), and plaque assays confirmed a significant reduction in viral titers, with an IC_50_ of 4.664 ± 1.15 µg/mL (Figure 5B). Based on the CC_50_ value, the SI against SHRV was calculated to be 81.64, indicating potent antiviral activity. These results demonstrate that the antiviral effects of the PL extract are not limited to rVHSV but extend to closely related rhabdoviruses, suggesting a mechanism targeting conserved features of rhabdoviral infection. However, as these findings are derived from surrogate and fish virus models, they do not establish antiviral activity across diverse mammalian RNA viruses. Therefore, the observed effects should be interpreted as evidence of antiviral activity within this viral group, with potential relevance to other enveloped RNA viruses sharing similar replication strategies. Further validation in mammalian virus systems will be required to confirm broader applicability.

## 5. Conclusions

This study demonstrates that the leaf extract of *P. amurense* (PL extract) exhibits potent antiviral activity against recombinant viral hemorrhagic septicemia virus (rVHSV) in EPC cells with low cytotoxicity. Mechanistic analyses indicate that the extract primarily targets early stages of infection, including virucidal and pre-treatment conditions, suggesting interference with viral entry or direct inactivation of viral particles. Solvent fractionation further revealed that antiviral activity is enriched in the n-hexane fraction, indicating that lipophilic, low-polarity compounds are likely responsible for the observed effects and contribute to improved selectivity compared with the crude extract. However, the specific bioactive compounds, their precise molecular mechanisms, and their potential synergistic effects among fractions remain to be elucidated. In addition, the PL extract showed strong inhibitory activity against snakehead rhabdovirus (SHRV), supporting its potential cross-reactivity against related viruses. Collectively, these findings identify *P. amurense* leaf extract as a promising source of antiviral compounds and support further studies to isolate active constituents and elucidate their molecular mechanisms, with potential applications in the development of broad-spectrum antivirals targeting enveloped RNA viruses.

## Figures and Tables

**Table 1:** Primers used for quantitative real-time PCR (qPCR).

Target Gene	Sequence (5′-3′)	Product Length (bp)	Reference
VHSV N	F-ATCTGGAGGCAAAGTGCAAG	138	[28]
	R-CCATGAGGTTGTCGTTGTTG		
B-actin	F-ATGTTCGAGACCTTCAACACCC	135	[29]
	R-CTCGTAGATGGGCACGGT		

**Table 2:** CC_50_, IC_50_, and selectivity index (SI) of solvent fractions of PL extract against rVHSV.

Sample	CC_50_ (µg/mL)	IC_50_ (µg/mL)	SI
crude extract	295.3	16.3	18.1
n-hexane fraction	110.0	3.1	35.5
ethyl acetate fraction	NE *	>200	NE *
n-butanol fraction	NE *	>200	NE *
aqueous fraction	NE *	>200	NE *

CC_50_, IC_50_, and SI values are presented to one decimal place. * NE; not evaluated because no antiviral activity was observed.

## Data Availability

The original contributions presented in this study are included in the article/Supplementary Material. Further inquiries can be directed to the corresponding author.

## Supplementary Materials

The following supporting information can be downloaded at: https://www.mdpi.com/article/10.3390/pathogens15050491/s1, Figure S1: Antiviral activity of the crude *P. amurense* leaf extract (PL extract) used for solvent fractionation against rVHSV. Figure S2: Cytotoxicity of the crude *P. amurense* leaf extract (PL extract) and its n-hexane fraction in EPC cells.
