# Supplementary material for: Phellodendron amurense Leaf Extract Inhibits Rhabdovirus Infection by Targeting Early Stages of Viral Entry
Source: Pathogens. 2026 May 1;15(5):491. doi: 10.3390/pathogens15050491 (PMC13210346; doi:10.3390/pathogens15050491)
Supplement: Supplementary file 1 [file pathogens-15-00491-s001.zip › pathogens-4257986-supplementary.pptx]

## Slide 1
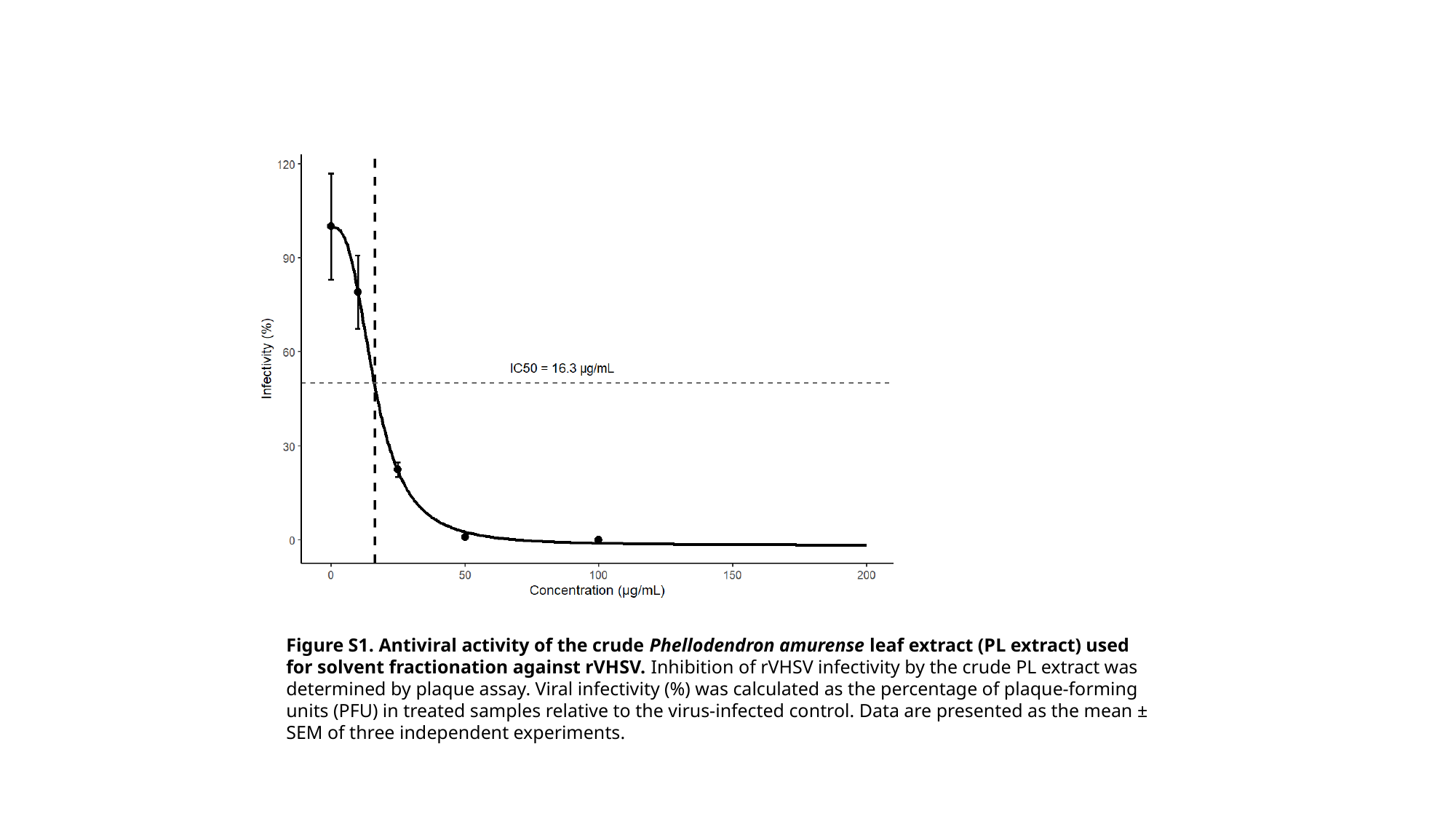

Figure S1. Antiviral activity of the crude Phellodendron amurense leaf extract (PL extract) used for solvent fractionation against rVHSV. Inhibition of rVHSV infectivity by the crude PL extract was determined by plaque assay. Viral infectivity (%) was calculated as the percentage of plaque-forming units (PFU) in treated samples relative to the virus-infected control. Data are presented as the mean ± SEM of three independent experiments.

## Slide 2
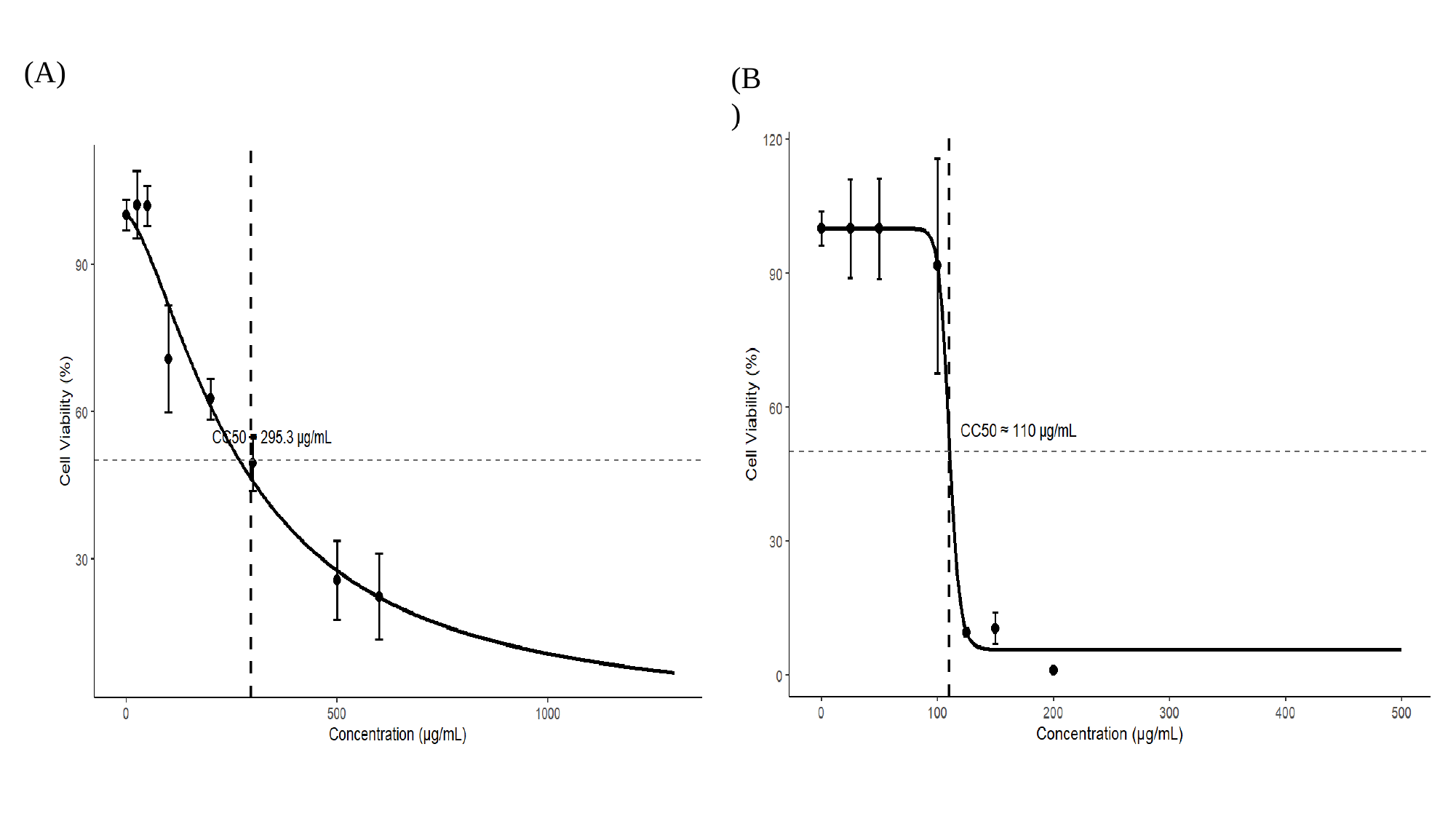

(A)
(B)
